# Leptin attenuates the osteogenic induction potential of BMP9 by increasing β-catenin malonylation modification via Sirt5 down-regulation

**DOI:** 10.18632/aging.205790

**Published:** 2024-05-03

**Authors:** Kai-Xin Ke, Xiang Gao, Lu Liu, Wen-Ge He, Yue Jiang, Cheng-Bin Long, Gan Zhong, Zheng-Hao Xu, Zhong-Liang Deng, Bai-Cheng He, Ning Hu

**Affiliations:** 1 Department of Pharmacology, School of Pharmacy, Chongqing Medical University, Chongqing 400016, People’s Republic of China; 2 Key Laboratory of Biochemistry and Molecular Pharmacology of Chongqing, Chongqing Medical University, Chongqing 400016, People’s Republic of China; 3 Department of Orthopaedics, The second affiliated hospital of Chongqing Medical University, Chongqing 400016, People’s Republic of China; 4 Department of Orthopaedics, The first affiliated hospital of Chongqing Medical University, Chongqing 400016, People’s Republic of China; 5 Department of Orthopaedics, Bishan Hospital of Chongqing Medical University, Chongqing 400016, People’s Republic of China

**Keywords:** BMP9, leptin, sirt5, β-catenin, lysine malonylation

## Abstract

BMP9 has demonstrated significant osteogenic potential. In this study, we investigated the effect of Leptin on BMP9-induced osteogenic differentiation. Firstly, we found Leptin was decreased during BMP9-induced osteogenic differentiation and serum Leptin concentrations were increased in the ovariectomized (OVX) rats. Both *in vitro* and *in vivo*, exogenous expression of Leptin inhibited the process of osteogenic differentiation, whereas silencing Leptin enhanced. Exogenous Leptin could increase the malonylation of β-catenin. However, BMP9 could increase the level of Sirt5 and subsequently decrease the malonylation of β-catenin; the BMP9-induced osteogenic differentiation was inhibited by silencing Sirt5. These data suggested that Leptin can inhibit the BMP9-induced osteogenic differentiation, which may be mediated through reducing the activity of Wnt/β-catenin signalling via down-regulating Sirt5 to increase the malonylation level of β-catenin partly.

## INTRODUCTION

BMP9, a member of the BMP family, has an attractive osteogenic induction potential than BMP7 or BMP2 in MSCs [1, 2]. It promotes osteogenic MSCs differentiation through multiple pathways [3]. Besides, BMP9 has a wide range of physiological functions. As reported, BMP9 is closely associated with liver disease, inflammatory response, and neurodevelopment [4–6]; but most importantly, BMP9 is involved in regulating the fate of MSCs. It’s well known that Wnt/β-catenin signalling is indispensable for osteogenesis, and can be initiated during the early stage of osteogenic differentiation but suppressed during the mature stage of osteoblast [7]. Wnt/β-catenin regulation is subject to a range of factors [8]. β-catenin plays a vital role in Wnt/β-catenin signal transduction and is subject to regulation by various post-translational modifications, including phosphorylation and acetylation [9]. BMP9 activates Wnt/β-catenin signalling when inducing precursor cells differentiate to the osteoblastic lineage [7]. However, how BMP9 affects the Wnt/β-catenin signalling activity has not been fully elucidated.

Leptin, a polypeptide made up of 167 amino acids, is predominantly expressed in white adipose tissue, bone marrow, and other tissues [10]. It was initially discovered in genetically obese rats, and the plasma Leptin affects food intake and energy balance by binding with its receptor [11, 12]. A previous report showed that a high level of glucose or Leptin inhibits the glycolytic process by promoting the secretion of malonyl coenzyme, a negative regulator of glucose metabolism which can promote critical regulators malonylation in the glycolytic pathway [13, 14]. During bone remodelling, Leptin is capable of regulating the equilibrium between bone formation and resorption through the stimulation of osteogenic or lipogenic differentiation. However, the actual mechanism remains unclear. Glycolysis is the main energy source during osteogenic differentiation [14], and can be regulated by Leptin through promoting the secretion of malonyl coenzyme A. Thus, Leptin may play a role in regulating the osteogenic differentiation of MSCs induced by BMP9.

Sirt5, an NAD+ dependent protein deacylase and located in the cytoplasm and mitochondria, is involved in regulating various metabolic pathways. It can regulate the glycolytic pathway by removing malonyl groups from the lysine residues of the target [15]. Malonyl coenzyme A, a donor of malonylated compounds produced by acetyl coenzyme A via acetyl coenzyme A decarboxylase, can be metabolized by malonyl coenzyme A decarboxylase [15]. Malonyl coenzyme A serves as a negative feedback regulator of metabolism by promoting the malonylation of targets. In contrast, Sirt5 can positively regulate metabolism by restoring the activity of related targets via demalonylation. Sirt5, as a global regulator of lysine malonylation, plays an important role in diseases associated with malonyl coenzyme A homeostasis [15]. However, its role in osteogenic differentiation has not been reported.

In this study, we assessed the effect of Leptin on osteogenic differentiation induced by BMP9, and determined whether Wnt/β-catenin and Sirt5 were both involved in this process.

## MATERIALS AND METHODS

### Reagents and cells

C3H10T1/2, HEK293, 3T3-L1 and MC3T3-E1 were obtained from ATCC (Manassas, VA, USA). DMEM, containing streptomycin, penicillin and 10% fetal bovine serum, was used for cell culture. OPN (sc-21742), Runx2 (sc-390715), BMP9 (sc-514211) from Santa Cruz (Shanghai, China), β-actin (AC038), Leptin (A1300), and Sirt5 (A5784) from Abclonal (Wuhan, China), and lysine malonylation antibody (PTM-902) were purchased from Jing Jie Biotechnology (Hangzhou, China).

### Rat ovariectomy (OVX) model

SD rats (female, 220~240 g, 6~8 weeks, 5/group) were anesthetized intraperitoneally with pentobarbital sodium. The rats were immobilized and disinfected. The skin was affixed to the superior border of the left femoral root. A 0.5 cm incision was made along the midline of the back. Milky white, shiny fat was identified through a thin muscle layer. The fat mass adjacent to the inferior pole of the kidney was considered the ovarian specimen. The superficial muscular layer of cellulite was lifted with forceps along the psoas major muscle. The lateral margin was cut approximately 0.5 cm and the left ovary was found. After the ovary was removed, the fallopian tubes were ligated with 5-0 silk thread. The right ovary was removed by the same procedure. After surgery, the surgical wound was sutured.

### Generation of adenovirus vectors expressing RFP, GFP, Leptin, β-catenin and BMP9

Adenovirus vectors utilized in this work were generated using the AdEasy system [16]. The coding sequences for mouse Leptin, β-catenin and BMP9 were amplified by PCR. The PCR products and the siRNA oligonucleotides for Leptin and β-catenin were cloned into a shuttle vector, separately; the shuttle vectors were then linearized with EcoRI (MF00801S; Monad, Suzhou, China) and transfected into BJ/5183 cells for homologous recombination. HEK293 cells were used for packaging recombinant viruses. Green fluorescent protein was added to AdBMP9, and red fluorescent protein to AdLeptin and AdsiLeptin. The viral titers of AdBMP9+, AdBMP9++ and AdBMP9+++ were MOI 10, MOI 15, MOI 20. The sequence for siRNA is shown in Table 1.

**Table 1:** Sequences for siRNA oligonucleotides.

**Gene**	**Accession No.**	**Sequence (5′→3′)**
Leptin	NM_008493.3	CCAAUGACCUGGAGAAUCU
		AGAUUCUCCAGGUCAUUGG
		UCCAGAAAGUCCAGGAUGA
		UCAUCCUGGACUUUCUGGA
		GGACUUCAUUCCUGGGCUU
		AAGCCCAGGAAUGAAGUCC
β-catenin	NM_007614.3	CCAGGUGGUAGUUAAUAAA
		UUUATTAACAACCACCUGG
		UAACCUCACUUGCAAUAAU
		AUUATTGCAAGTGAGGUUA
		CUAUCAGGAUGACGCGGAA
		UUCCGCGUCAUCCUGAUAG

### RNA preparation and qRT-PCR

TRIzol reagent (15596018, Ambion, Austin, TX, USA) was used for RNA extraction from cells. Next, 1 μg RNA product was utilized for reverse transcriptase (RT) reaction to produce cDNA with RT kit (R037A; Takara, Beijing, China). cDNA samples were mixed with 2X SYBR Green mix kit. Bio-Rad CFX Connect system (Bio-Rad, Chicago, IL, USA) was used for real-time PCR assay. Primers used for this study are shown in Table 2.

**Table 2:** The primers used for PCR assay.

**Gene**	**Primer**	**Sequence (5′ →3′)**
Leptin	F	GGATCAGGTTTTGTGGTGCT
	R	TTGTGGCCCATAAAGTCCTC
Runx2	F	GCCAATCCCTAAGTGTGGCT
	R	AACAGAGAGCGAGGGGGTAT
OPN	F	TGCACCCAGATCCTATAGCC
	R	CTCCATCGTCATCATCATCG
β-actin	F	CCACCATGTACCCAGGCATT
	R	CGGACTCATCGTACTCCTGC

F, forward; R, reverse.

### Western blot assay

Cells were lysed in RIPA (QS0006, Saimike Biotech, Chongqing, China) with protease and phosphatase inhibitors. Proteins were separated by 10% SDS-PAGE and then transferred to PVDF membranes. The primary antibodies were used for incubation overnight at 4° C, secondary antibodies conjugated with horseradish peroxidase were used for further incubation (30 min). Proteins were detected by chemiluminescence using the ChemiScope 6200 (Clinx, Shanghai, China). The data were analyzed with ImageJ software (Version 1.53e, NIH, Bethesda, MD, USA).

### Alkaline phosphatase staining

Cells were stained with Alkaline phosphatase staining kit (C3206, Beyotime Biotech, Haimen, China) following the instructions. Images were taken with a microscope. ImageJ (Version 1.53 e, NIH, Bethesda, MD, USA) was used for quantitative analysis.

### Mouse cranial defect repair assay

The cranial defect model was established as previously reported [17]. Briefly, cranial defect surgery was performed on 6~8 weeks old mice (female, 20~24 g, 5 mice per group). Hair of the surgical site was removed from the skull. 3 mm diameter trephine was used to establish cranial defect. Pre-treated cells were transferred to the defect areas. Eight weeks later, all mice were euthanized, the crania were collected, and fixed with formalin for the further evaluation.

### Micro-computed tomography (μ-CT) assay

A vivaCT 40 *in vivo* preclinical micro-CT scanner system (SCANCO Medical AG, Wangen-Brüttisellen, Switzerland) was used to scan the cranial defect repair samples. Raw data were quantitatively analyzed, and 3D reconstructions were performed with CT 516.1 software.

### LC-MS analysis

LC-MS was performed to identify the malonylation proteins. In briefly, cells were harvested on ice and incubated with anti-malonylation antibody, and then treated with protein A/G magnetic beads (B23202, Bimake, USA). Samples were quickly run on an SDS-PAGE gel. The dissolved gel slices were subjected to TripleTOF 6600, and ProteinPilot™ was used for identification. R package (clusterProfiler) was used to perform KEGG enrichment analyses of malonylated proteins.

### ELISA assay

Serum tartrate-resistant acid phosphatase (TRAP), osteocalcin (OCN), and Leptin in OVX rats were measured using ELISA kits. A spectrophotometer (Varioskan 3020, Thermo Fisher Scientific, Waltham, MA, USA) was used to measure the optical absorbance at 450 nm.

### Confocal analysis

Cells were fixed with 4% polyformaldehyde, and then incubated with PBS plus 0.3% Triton X-100, blocked with BSA. Then, cells were incubated with primary antibody overnight at 4° C and incubated with secondary antibody conjugated with DyLight 594 (ab96881, Abcam, Shanghai, China) for 30 min. Finally, cells were incubated with diamino phenylindole (DAPI) for 1 min. Images were taken with confocal microscope (SP8, Leica, Germany).

### IP assay

Cells were lysed in IP lysis buffer (20 mM Tris-HCl, 150 mM NaCl, 1 mM EDTA, 1% Triton X-100) with protease/phosphatase inhibitors (B15001, Bimake, Houston, TX, USA). The lysates were pretreated with Protein A/G magnetic beads (P2108-1 ml, Beyotime Biotech, Haimen, China), and incubated with primary antibody against β-catenin, lysine malonylation, Sirt5, or rabbit IgG at 4° C overnight. The precipitates were rinsed carefully with RIPA lysis buffer twice, and the precipitates were boiling for 10 min to elute the proteins with RIPA lysis buffer. Finally, Western blot assay was used for further detection.

### Statistical analysis

Data were analyzed using Prism 6 (GraphPad) as mean ± SD. Tukey's post hoc test or two-tailed Student's *t*-test was used for one-way analysis of variance to evaluate differences. The *P-*value less than 0.05 was considered to indicate statistically significant differences.

### Availability of data and materials

The data and materials supporting the conclusions of this article are available upon request.

## RESULTS

### Effects of ovariectomy on the serum levels of Leptin in SD rats

**Figure 1:**
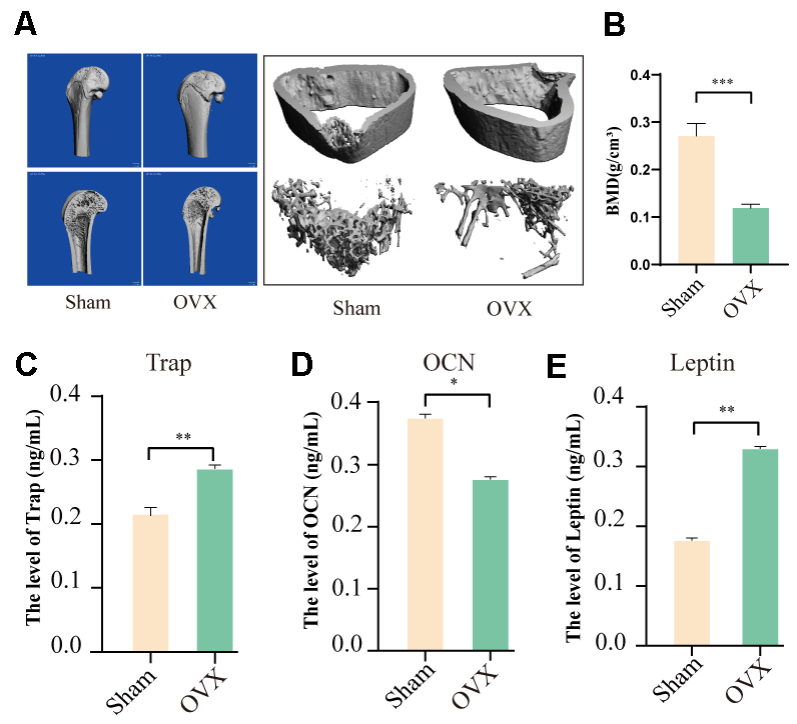
**The effects of ovariectomy on bone formation and Leptin levels in OVX SD rats.** (**A**) Micro-CT scanning results of femurs from the OVX and sham SD rats. (**B**) Quantitative analysis of bone parameters. Bone mineral density (BMD). (**C**–**E**) ELISA assay results show the levels of Trap, OCN, and Leptin in the serum of OVX and sham rats. (“*” *P*<0.05, “**” *P* < 0.01).

There is increasing evidence that postmenopausal osteoporosis may result from abnormal energy metabolism [18]. Thus, the imbalance between bone formation and resorption may due to the dysfunctional energy metabolism [19, 20]. Leptin was reported to be involved in balancing bone formation and resorption by regulating energy metabolism, but the specific mechanism of this process remains unclear. In this study, we firstly established an OVX model, micro-CT results showed the OVX model was successfully constructed (Figure 1A, 1B). Subsequently, Serum levels of TRAP, OCN, and Leptin in OVX rats were measured using ELISAs. Results showed a significant reduction in OCN levels in the OVX group, but a significant increase in TRAP and Leptin levels (Figure 1C–1E). These results suggest that Leptin may play a role in regulating bone formation.

### Effects of BMP9 on Leptin in C3H10T1/2 cells

**Figure 2:**
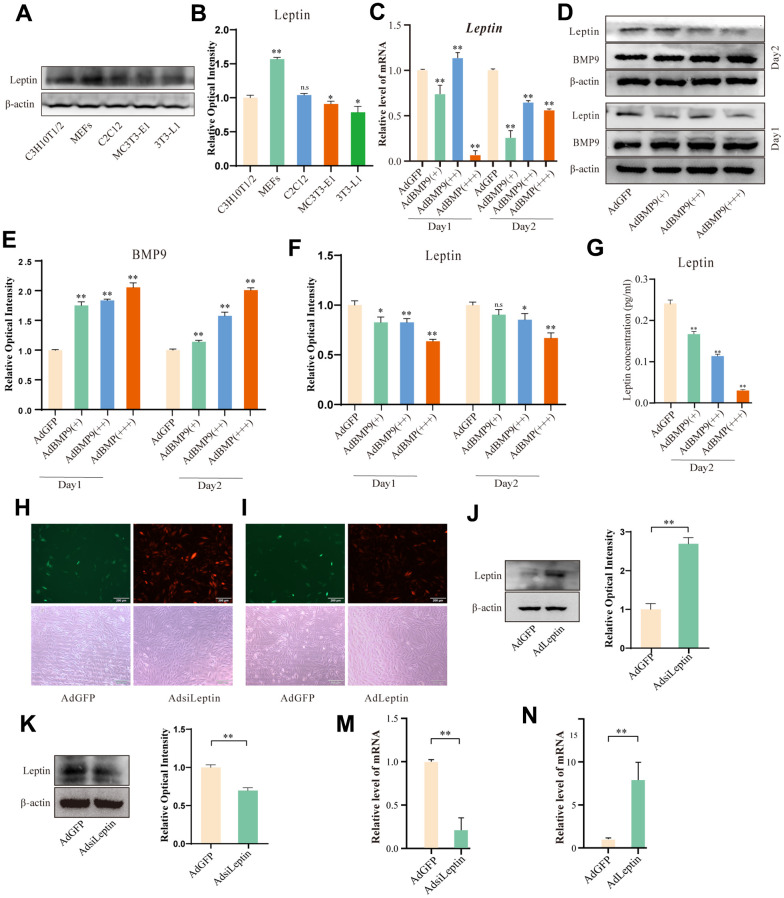
**The effects of BMP9 on Leptin in C3H10T1/2 cells.** (**A**) Endogenous Leptin levels in multipotent progenitor cells. (**B**) Semi-quantification of the level of Leptin. (**C**) qPCR assay shows the effect of BMP9 on Leptin. (**D**) Western blot assay shows the effect of BMP9 on Leptin. (**E**, **F**) Semi-quantification of the level of BMP9 or Leptin. (**G**) ELISA assay shows the effect of BMP9 on the concentration of Leptin in culture medium. (**H**, **I**) Images show the transduction of recombinant adenovirus in C3H10T1/2 cells. (**J**–**N**) Western blot and qPCR assay show the effect of recombinant adenovirus on Leptin (48 h). The scale bar is 200 μm. (“*” *P*<0.05, “**” *P* < 0.01).

Next, we measured Leptin levels in several progenitor cell lines and the effect of BMP9 on Leptin. Western blot showed that Leptin was lower in C3H10T1/2 (Figure 2A, 2B). Because the C3H10T1/2 has been widely used for research on osteogenic or lipogenic differentiation, so we selected C3H10T1/2 for subsequent research. Then, we examined the relationship between BMP9 and Leptin. qPCR and Western blot assay results showed that BMP9 significantly reduced the expression of Leptin (Figure 2C–2F). ELISA assay results demonstrated that BMP9 also caused a decrease in the level of Leptin present in the culture medium (Figure 2G). These data suggested that Leptin may be involved in BMP9-induced osteogenic differentiation. Then, we constructed recombinant adenovirus for overexpressing or silencing mouse Leptin using the AdEasy system (Figure 2H, 2I). The functions of recombinant adenovirus were confirmed with Western blot and qPCR assay (Figure 2J–2N).

### The effects of Leptin on BMP9-induced osteogenic differentiation

**Figure 3:**
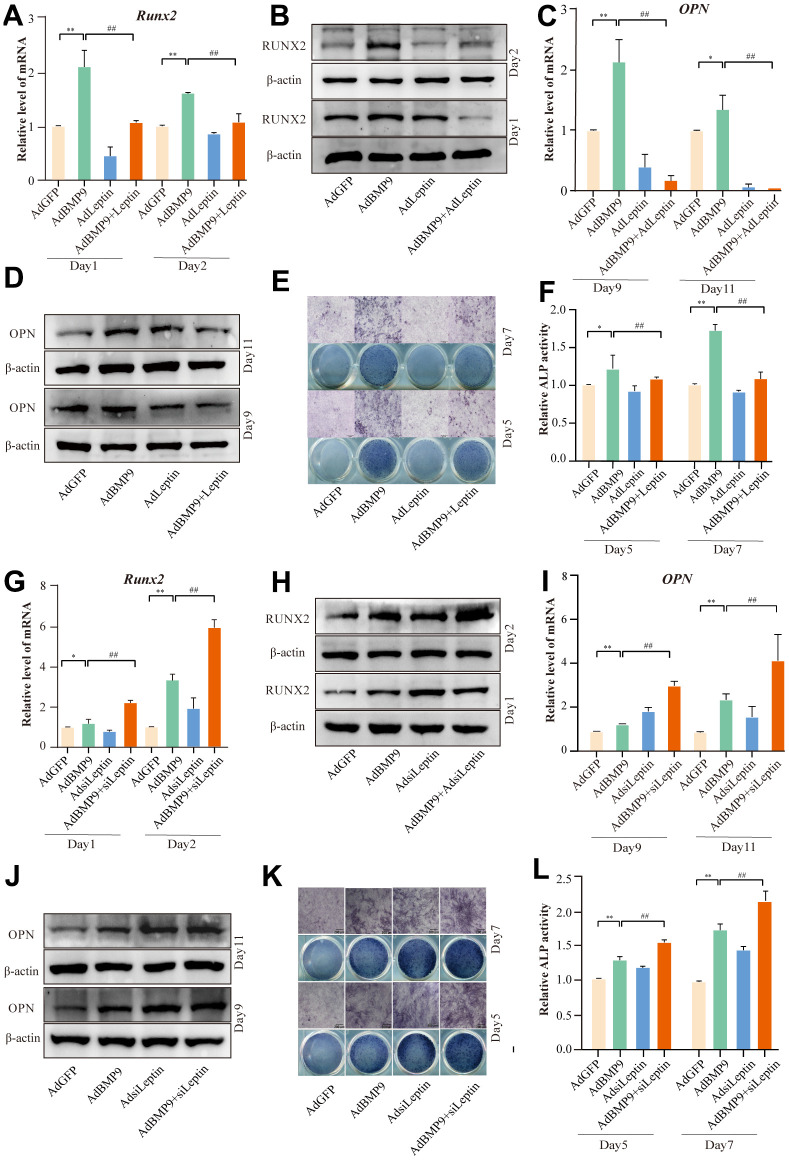
**The effects of Leptin on BMP9-induced osteogenic markers in C3H10T1/2 cells.** (**A**–**D**) qPCR and Western blot assay shows the effect of overexpressing Leptin on Runx2 and OPN. (**E**) ALP staining shows the effect of overexpressing Leptin on ALP activity. (**F**) Quantitative result of ALP staining. (**G**–**J**) qPCR and Western blot assay show the effect of silencing Leptin on Runx2 and OPN. (**K**) ALP staining shows the effect of silencing Leptin on ALP activity. (**L**) Quantitative results of ALP staining. The scale bar is 200 μm. (“*” *P* < 0.5, “**” *P*< 0.01; “#” *P*<0.05, “##” *P* < 0.01).

To further elucidate the function of Leptin in BMP9-induced osteogenic differentiation. Firstly, we found that AdLeptin could reverse the downregulation of Leptin caused by BMP9 (Supplementary Figure 1A). Next, the osteogenic-related producers were examined. qPCR and Western blot showed that the level of Runx2 and OPN were decreased by over-expressing Leptin, (Figure 3A–3D and Supplementary Figure 1B, 1C). Similar results were found in ALP activity (Figure 3E, 3F). In contrast, the level of Runx2 OPN, ALP activity was increased by silencing Leptin (Figure 3G–3L and Supplementary Figure 1D, 1E). These results suggested that Leptin may be a negative regulator of BMP9-induced osteogenic differentiation.

### Effects of BMP9 and/or Leptin on Wnt/β-catenin

**Figure 4:**
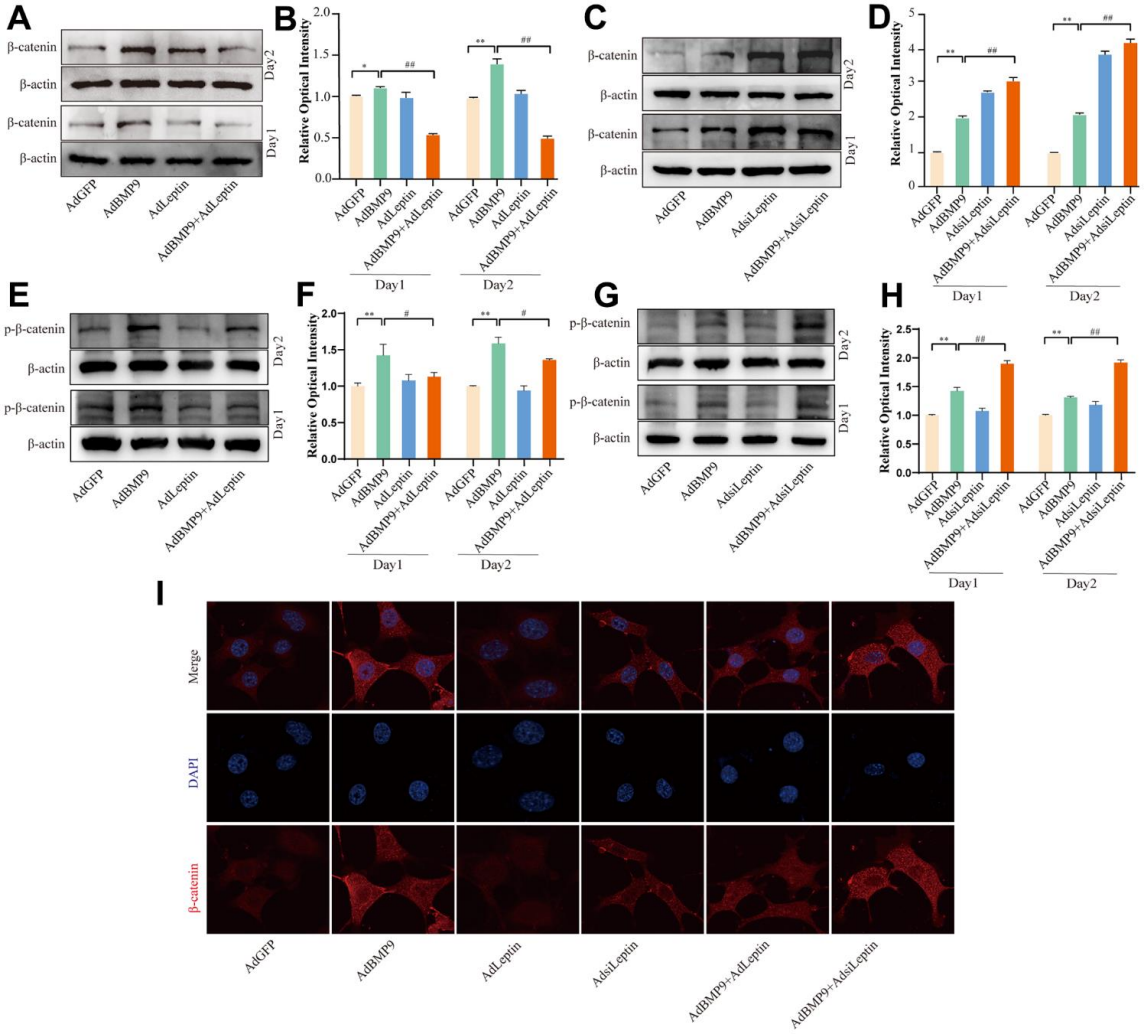
**The effects of BMP9 and/or Leptin on Wnt/β-catenin in C3H10T1/2 cells.** (**A**) Western blot assay shows the effect of overexpressing Leptin on β-catenin. (**B**) Semi-quantification of the level of β-catenin. (**C**) Western blot assay shows the effect of knocking down Leptin on β-catenin. (**D**) Semi-quantification of the level of β-catenin. (**E**) Western blot assay shows the effect of overexpressing Leptin on phosphorylated β-catenin. (**F**) Semi-quantification of the level of phosphorylated β-catenin. (**G**) Western blot assay shows the effect of knocking down Leptin on phosphorylated β-catenin. (**H**) Semi-quantification of the level of phosphorylated β-catenin. (**I**) Confocal assay shows the effect of Leptin on β-catenin. (“*” *P* < 0.5, “**” *P* < 0.01 vs. the control; “#” *P*< 0.05, “##” *P* < 0.01).

Wnt/β-catenin is an important signalling pathway in BMP9-induced osteogenic differentiation, but it remains unclear how BMP9 facilitates this signalling pathway. Activation of Wnt/β-catenin signalling was found to promote aerobic glycolysis and potentially accelerate osteogenic differentiation of MSCs [21]. Leptin regulates metabolic activity by promoting the secretion of malonyl coenzyme A, which may also affect the Wnt/β-catenin signalling. Thus, we next determined whether Leptin could affect this pathway. Western blot showed that Leptin decreased the level of β-catenin (Figure 4A, 4B). In contrast, the level of β-catenin was increased by silencing Leptin (Figure 4C, 4D). We also examined phosphorylated beta-catenin (Ser675) levels and revealed the same results (Figure 4E, 4H). The confocal assay results also showed the same results (Figure 4I). These data suggested that the effect of Leptin on BMP9-induced osteogenic differentiation may be mediated in part by reducing Wnt/β-catenin signalling.

### Effects of β-catenin and/or Leptin on BMP9-induced osteogenic differentiation and bone reconstruction

**Figure 5:**
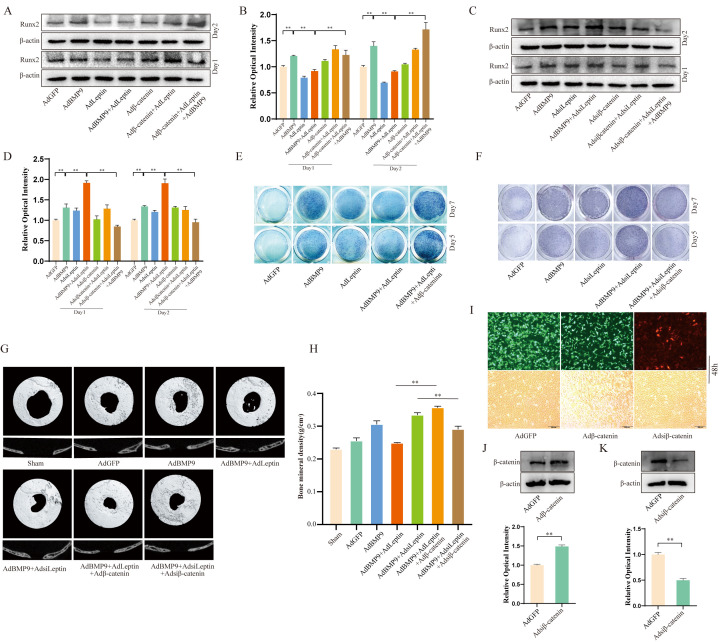
**The effects of Leptin and/or β-catenin on BMP9-induced osteogenic markers and bone defect repair.** (**A**) Western blot assay shows the effect of overexpressing Leptin and/or β-catenin on Runx2. (**B**) Semi-quantification of the level of Runx2. (**C**) Western blot assay shows the effect of knocking down Leptin and/or β-catenin on Runx2. (**D**) Semi-quantification of the level of Runx2. (**E**) ALP staining shows the effect of overexpressing Leptin and/or β-catenin effect on ALP activities. (**F**) ALP staining shows the effect of knocking down Leptin and/or β-catenin on ALP activities. (**G**) The 3D reconstruction of μ-CT scanning data shows the effect of β-catenin and/or Leptin on BMP9-induced bone repair (representative data are shown). (**H**) Quantitative analysis of μ-CT scanning (bone mineral density). (**I**) Images show the infection efficiency of AdGFP, Adβ-catenin, and Adsiβ-catenin in C3H10T1/2 cells (48 h). (**J**, **K**) Western blot assays show the effect of recombinant adenovirus on β-catenin in C3H10T1/2 cells (48 h). The scale bar is 200 μm. (“*” *P* < 0.05, “**” *P* < 0.01; “#” *P* < 0.05, “##” *P* < 0.01).

Since β-catenin can be negatively regulated by Leptin, next, we determined whether the over-expression of β-catenin could reverse the effects of Leptin on BMP9. Western blot showed that the over-expression of Leptin reduced the osteogenic differentiation induced by BMP9, which was partially reversed by over-expression of β-catenin (Figure 5A, 5B), In contrast, the effect of silencing Leptin on BMP9-induced osteogenic markers was almost eliminated by silencing β-catenin (Figure 5C, 5D); the same results were observed with ALP staining (Figure 5E, 5F). We also used the cranial defect repair model to evaluate the effect of Leptin and/or β-catenin on BMP9-induced osteogenic differentiation. The results showed the BMP9 potential to induce bone defect repair was attenuated by over-expressing Leptin, and was almost reversed by over-expressing β-catenin. In contrast, BMP9-induced osteogenic differentiation was enhanced by silencing Leptin, and almost eliminated by silencing β-catenin (Figure 5G, 5H). The functions of recombinant adenovirus were demonstrated by Western blot (Figure 5I–5K). These results indicate Leptin may inhibit the BMP9 osteogenic potential via reducing the activity of Wnt/β-catenin signalling.

### Effects of BMP9 on malonylation of β-catenin

**Figure 6:**
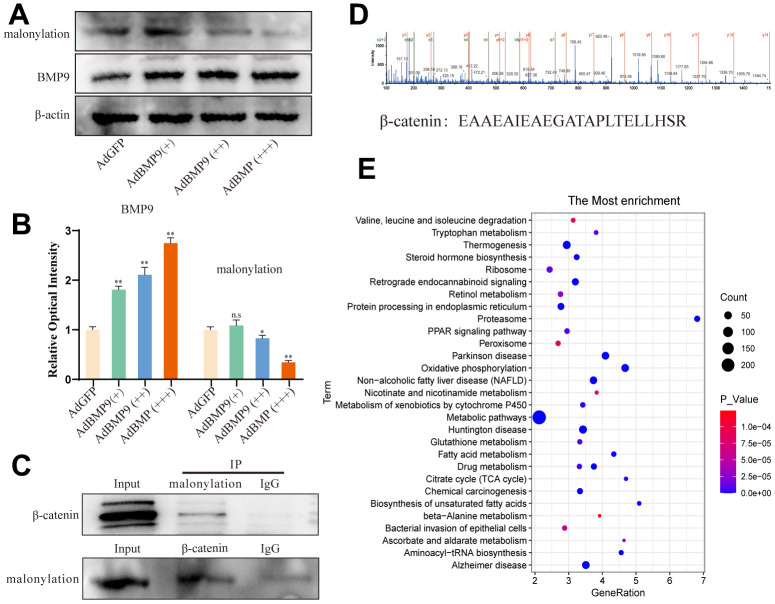
**The effects of BMP9 on malonylation of β-catenin in C3H10T1/2 cells.** (**A**) Western blot assay shows the effect of BMP9 on lysine malonylation. (**B**) Semi-quantification of the level of BMP9 or malonylation. (**C**) IP assay shows the lysine malonylation of β-catenin. (**D**) LC–MS analysis shows the malonylation proteins in C3H10T1/2. (**E**) KEGG pathway analysis shows the possible biochemical processes or diseases that may be associated with lysine malonylation. (“*” *P* < 0.05, “**” *P* < 0.01).

Leptin can regulate metabolism by promoting the secretion of malonyl coenzyme A. This coenzyme acts as a donor for malonylation, and inhibits the activity of critical metabolic enzymes through post-translational modification. Thus, we determined whether the malonylation level could be affected by BMP9. We found that the malonylation level was decreased by BMP9 (Figure 6A, 6B). It suggested that malonyl modification may exhibit a negative effect on osteogenic differentiation. Therefore, we further investigated whether β-catenin could be modified with malonylation. The IP assay results showed that β-catenin can be modified with malonylation (Figure 6C). Further liquid chromatography-mass spectrometry analysis results showed that β-catenin can be modified with malonylation (Figure 6D). KEGG enrichment analysis showed that malonylation occurs mainly in metabolic pathways, fatty acid oxidation, the citrate cycle, and other pathways (Figure 6E). These data indicated that malonylation of β-catenin may play an important role in regulating BMP9-induced osteogenic differentiation.

### Effects of Sirt5 on Leptin-induced malonyl modification of β-catenin

**Figure 7:**
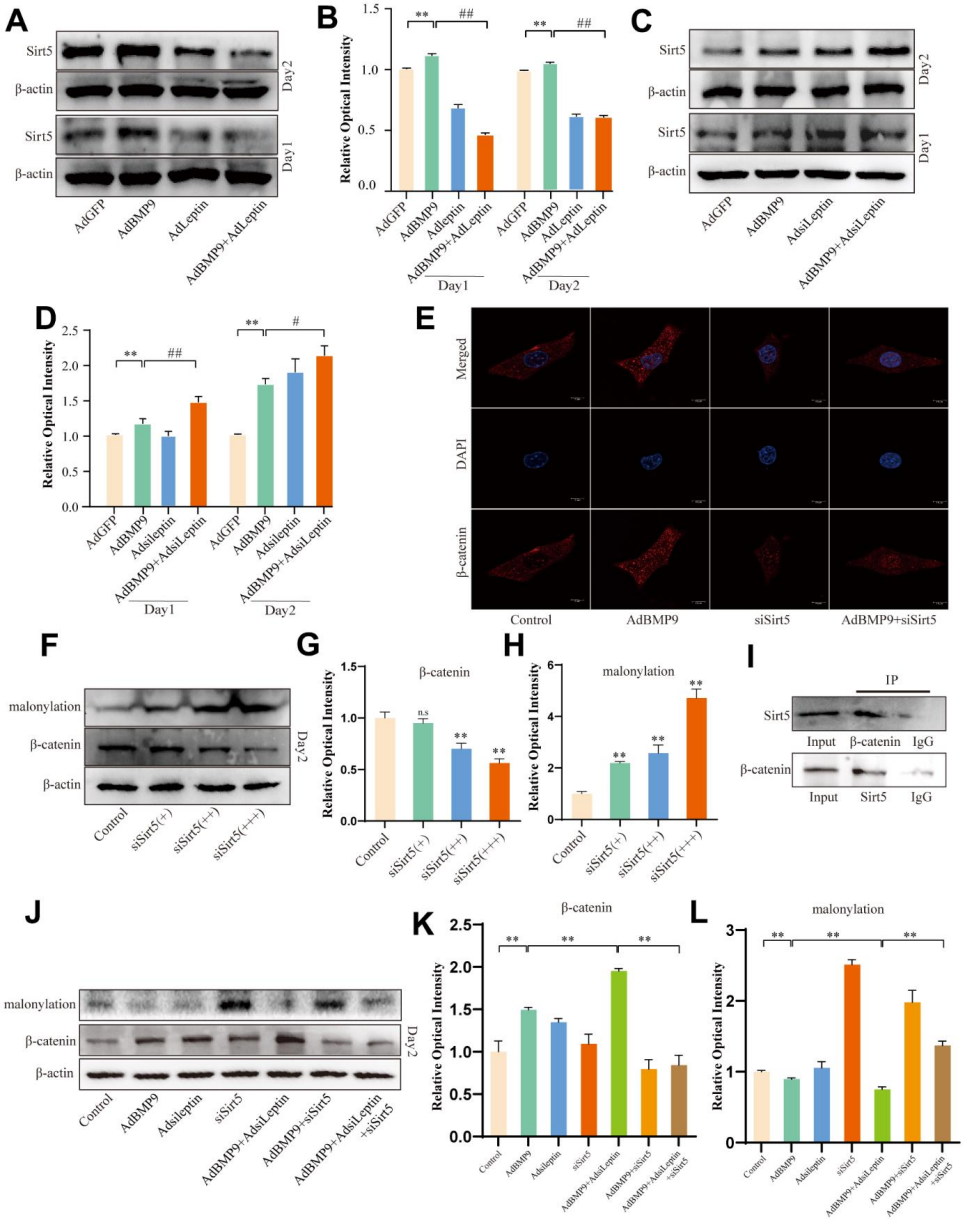
**The effects of Sirt5 on Leptin-induced malonyl modification of β-catenin.** (**A**) Western blot assay shows the effect of overexpressing Leptin on Sirt5. (**B**) Semi-quantification of the level of Sirt5. (**C**) Western blot assay shows the effect of knocking down Leptin on Sirt5. (**D**) Semi-quantification of the level of Sirt5. (**E**) Confocal assay shows the effect of knocking down Sirt5 on β-catenin. (**F**) Western blot assay shows the effect of knocking down Sirt5 on the level of lysine malonylation and β-catenin. (**G**, **H**) Semi-quantification of the level of β-catenin or malonylation. (**I**) IP assay shows the possible interaction between Sirt5 and β-catenin. (**J**) Western blot assay shows the effect of knocking down Sirt5 and/or Leptin on β-catenin and malonylation modification. (**K**, **L**) Semi-quantification of the level of β-catenin or malonylation. (“*” *P* < 0.05, “**” *P* < 0.01; “#” *P* < 0.05, “##” *P* < 0.01).

Since Sirt5 is a frequently observed demalonylase, we determined whether Leptin could regulate the malonylation of β-catenin via Sirt5. Western blot showed the over-expression of Leptin reduced the level of Sirt5 (Figure 7A, 7B). In contrast, Sirt5 was increased by silencing Leptin (Figure 7C, 7D). Since Leptin can reduce the level of β-catenin and its nuclear translocation [22, 23], we examined the effect of Sirt5 on β-catenin. Silencing Sirt5 reduced the level of β-catenin and its nuclear translocation as revealed by laser confocal microscopy analysis (Figure 7E). Western blot revealed an increase in malonylation levels and a decrease in β-catenin levels as the result of Sirt5 silencing (Figure 7F–7H). These results preliminarily confirmed that Leptin can regulate β-catenin via Sirt5. To further verify this hypothesis, we examined the relationship between Leptin/Sirt5 and malonylation modification. IP analysis results revealed an interaction between Sirt5 and β-catenin (Figure 7I). Further, Western blot showed that silencing Leptin increased the level of β-catenin and decreased its malonylation level, which were reversed by silencing Sirt5 (Figure 7J–7L). These results suggested that the Leptin effect on Wnt/β-catenin signalling may partially due to maintain the malonylation of β-catenin via down-regulating Sirt5.

### Effects of Sirt5 on BMP9-induced osteogenic differentiation

**Figure 8:**
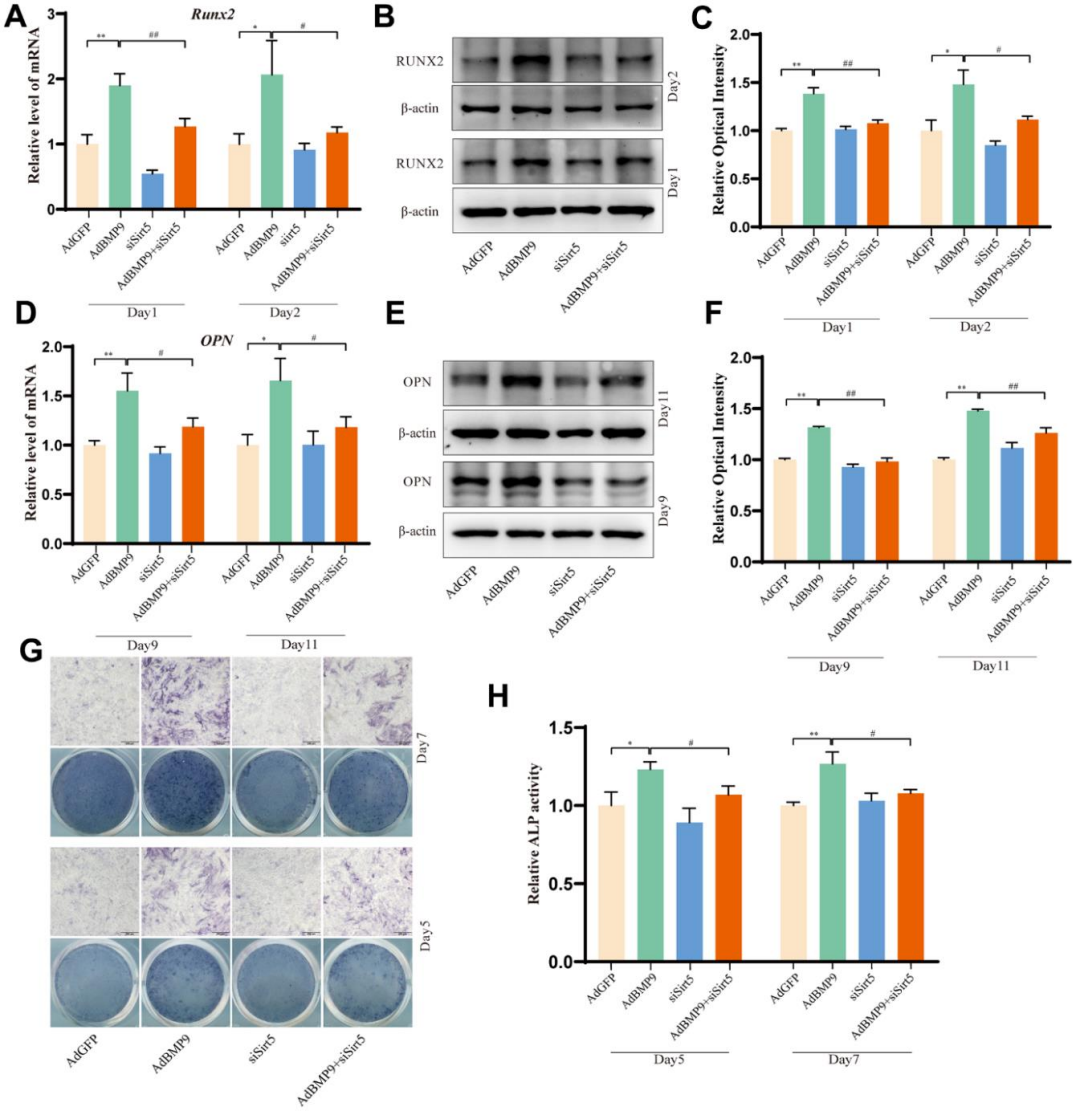
**The effects of Sirt5 on BMP9-induced osteogenic markers in C3H10T1/2 cells.** (**A**, **B**) qPCR and Western blot assay show the effect of knocking down Sirt5 on Runx2. (**C**) Quantification of the Western blot assay. (**D**, **E**) qPCR and Western blot assay show the effect of knocking down Sirt5 on OPN. (**F**) Quantification of the Western blot assay. (**G**) ALP staining shows the effect of knocking down Sirt5 on ALP activity. (**H**) Quantitative results of ALP staining. (“*” *P* < 0.5, “**” *P*< 0.01; “#” *P*<0.05, “##” *P* < 0.01).

Finally, we examined whether Sirt could affect the BMP9-induced osteogenic differentiation in progenitor cells. qPCR and Western blot assay results showed that levels of Runx2 and OPN were decreased by silencing Sirt5 in C3H10T1/2. (Figure 8A–8F). Similar results were found in ALP activity (Figure 8G, 8H). These results suggested that Sirt5 may strengthen the BMP9 osteogenic potential.

## DISCUSSION

BMP9 has excellent osteogenic potential and may be used as a promising candidate for bone tissue engineering. In addition to its excellent performance in bone repair, BMP9 also plays an important role in regulating bone metabolic diseases. It was found that BMP9 can reduce bone loss and improve bone biomechanical properties by increasing bone formation activity and inhibiting bone resorption activity in OVX rat model [24]. BMP9 often exerts the above function by activating Wnt/β-catenin signalling. At the same time, enhancement of Wnt/β-catenin signalling can further promote osteogenic differentiation. However, how BMP9 regulates this signalling remains unclear. In this study, we found that the BMP9-induced osteogenic differentiation can be inhibited by Leptin; the BMP9-induced activation of Wnt/β-catenin may be partially derived from Leptin down-regulation, which leads to up-regulation of Sirt5 and reduces the malonylation level of β-catenin.

Osteogenesis is a very complex physiological process which is coordinately regulated by multiple factors. BMP/Smad and Wnt/β-catenin signalling are important pathways for MSCs osteogenic differentiation [8]. The osteogenic potential of BMP was first discovered by Urist in 1965 [25], and BMP9 may be one of the strongest osteogenic factors in the BMP family [1]. In addition to BMP/Smad signalling, the BMP9 effect on osteogenic differentiation can also be mediated through noncanonical BMP/Smad signalling, such as Wnt/β-catenin, retinoic acid, and insulin-like growth factor 1 [1, 26]. Wnt/β-catenin is very important for skeletal development, and BMP9 can promote the transcription of Runx2 by promoting β-catenin nucleus translocation [27]. However, how BMP9 activates Wnt/β-catenin signalling is still kept unclear.

Leptin is mainly secreted by adipocytes and binds with its receptors in brain to activate the related signalling pathways, which results in food consumption inhibition and energy expenditure promotion [28]. In addition, Leptin participates in multiple endocrine regulatory functions, including immune and inflammatory responses, angiogenesis, bone formation, and wound healing [28]. Increasing evidence showed a close relationship between Leptin and osteogenesis [29]. Leptin can exert a direct effect on the anabolism of osteoblasts and chondrocytes, and regulate bone metabolism indirectly by affecting the production of other hormones, such as pituitary hormones [30]. By regulating the activity of acetyl-CoA carboxylase, leptin may promote the synthesis of malonyl-CoA, which is involved in the malonyl modification of various signalling regulatory proteins [31]. However, the specific mechanism for this process is still unclear. Leptin has been reported to inhibit osteogenesis, we found Leptin was a downstream protein of BMP9, and BMP9 can downregulate its expression in a concentration-dependent manner, which may be the potential mechanism of BMP9-induced osteogenic differentiation. The total malonylation level was reduced by BMP9, so did the expression of Leptin. Therefore, we hypothesized that Leptin may inhibit osteogenesis by regulating malonyl modification of some major osteogenic-related factors.

Malonyl-CoA, a precursor of the synthesis of fatty acids and a negative regulator of the oxidation of fatty acids [32], is synthesized mainly by acetyl-CoA carboxylase and conjugated with long-chain fatty acids [33]. In hypothalamus, malonyl-CoA fluctuates in response to physiological and/or hormonal stimulation, such as fasting, glucose, Leptin and thyroid hormone [32, 34, 35]. Malonyl-CoA was reported to regulate food intake and energy metabolism through post-translational modification. Malonylation modification is a novel post-translational modification which can not only change the protein structure but also inhibit its activity [32, 36]. For example, malonylation of glycerol 3 phosphate dehydrogenase, an important enzyme of the glycolytic pathway, can reduce its activity and function [37]. It has been reported that Leptin may regulate energy metabolism by affecting malonyl-CoA production. Therefore, we hypothesized that the Leptin effect on the osteogenic differentiation of MSCs may be mediated by regulating the malonyl modification of some critical factors in osteogenic pathways.

To date, there has been few reports on the effect of malonyl modification of osteogenic regulators. It’s well known that β-catenin is an integral structural component of cadherin-based adhesion and a critical effector of Wnt/β-catenin pathway in nucleus, and its modification and/or abnormal levels is associated with various diseases [38]. The Wnt/β-catenin signal transduction can be modulated by various factors [39, 40]. Multiple skeletal diseases are caused by aberrant Wnt/beta-catenin signalling, including tetra-amellia syndrome, autosomal recessive osteoporosis, pseudoglioma syndrome, and sclerosteosis [41, 42]. β-catenin plays an important role in metabolism, such as increasing the expression of key genes for fatty acid oxidation and mitochondrial β-oxidase [26, 43, 44]. Malonyl modification mainly occurs in metabolic pathways, such as fatty acid oxidation [45]. Up to now, there is no report about the malonyl modification of β-catenin. In this study, immunoprecipitation and mass spectrometry were used to explore the possible mechanism of Leptin in BMP9-induced osteogenic differentiation. We found that BMP9 reduced the levels of Leptin and malonylation, and β-catenin can be modified with malonylation; the malonylation level of β-catenin was reduced by BMP9 in C3H10T1/2 cells. Exogenous Leptin inhibited osteogenic differentiation and increased the malonylation of β-catenin. However, how BMP9 inhibits Leptin expression and reduces the level of β-catenin malonylation remains unclear.

Sirt5 is a member of the sirtuin family and serves as a global regulator of malonylation. To date, Sirt5 has already been defined as a new potential therapeutic target for the treatment of various diseases [46, 47]. The substrates of Sirt5-induced malonylation are involved in many biological processes, including glycolysis, TCA cycle, fatty acid oxidation, electron transport chain, nitrogenous waste management, and detoxification of reactive oxygen species [48]. However, the role of Sirt5 in osteogenic differentiation is unclear. In periapical periodontitis, the expression of Sirt5 was reduced along with an increase of oxidative stress and bone lining cell apoptosis, which could be reversed by exogenous Sirt5 [49]. Although the specific mechanism has not yet been clarified, these results suggested that Sirt5 may positively regulate osteogenic differentiation, which is consistent with our results. We found that Leptin can inhibit osteogenic differentiation along with down-regulation of Sirt5 and up-regulation of β-catenin malonylation. Moreover, we also found for the first time that Sirt5 knockdown decreased β-catenin protein levels, but increased its malonyl modification level. Therefore, Sirt5 may activate Wnt/β-catenin signalling through de-malonylation of β-catenin, and Leptin knockdown may activate Wnt/β-catenin signalling by promoting Sirt5 expression.

**Figure 9:**
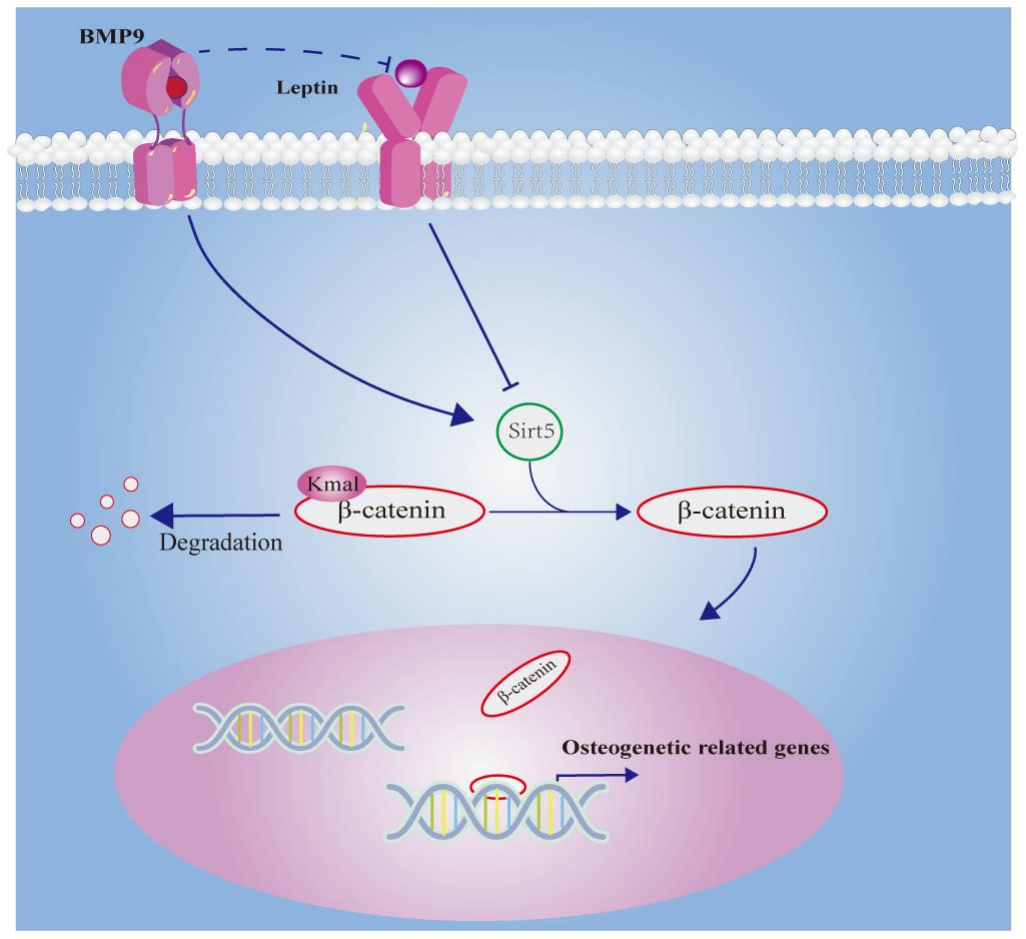
Schematic illustration of this work.

In summary, this study suggested that Leptin can inhibit osteogenic differentiation, which may be mediated by down-regulating Sirt5 to maintain the malonylation of β-catenin and inhibit the activity of β-catenin (Figure 9). In addition, our findings provide a potential new effective strategy to promote osteogenic differentiation, which may accelerate bone tissue engineering.

## Supplementary Material

Supplementary Figure 1
