# Supplementary material for: Leptin attenuates the osteogenic induction potential of BMP9 by increasing β-catenin malonylation modification via Sirt5 down-regulation
Source: Aging (Albany NY). 2024 May 3;16(9):7870–88. doi: 10.18632/aging.205790 (PMC11131982; doi:10.18632/aging.205790)
Supplement: Supplementary Figure 1 [file aging-16-205790-s001.pdf]

## SUPPLEMENTARY FIGURE

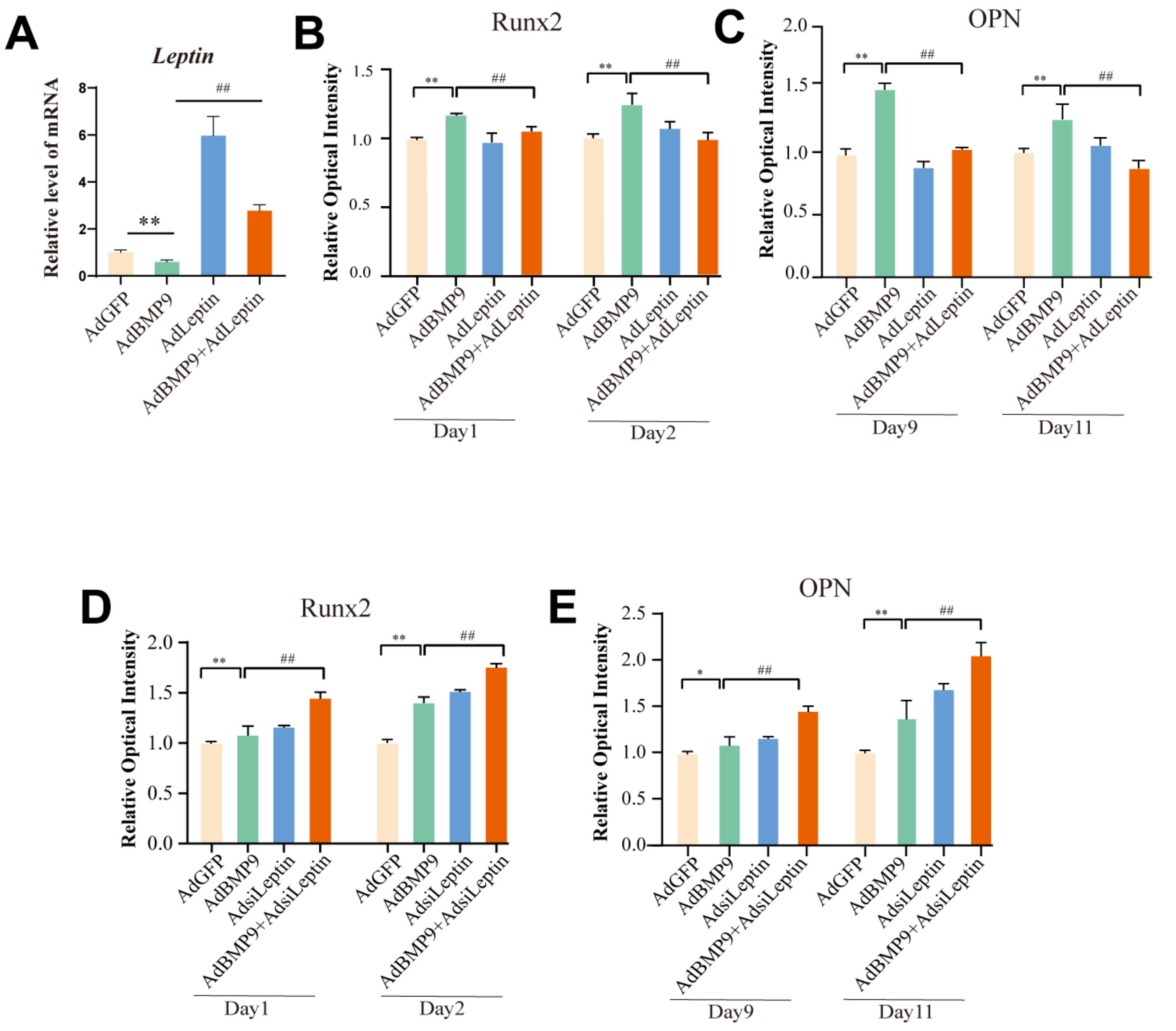


**Supplementary Figure 1.** (**A**) qPCR shows the level of Leptin. (**B**) Semi-quantification of the level of Runx2. (**C**) Semi-quantification of the level of OPN. (**D**) Semi-quantification of the level of Runx2. (**E**) Semi-quantification of the level of OPN.
